# More Is More: Drivers of the Increase in Emergency Medicine Residency Applications

**DOI:** 10.5811/westjem.2020.10.48210

**Published:** 2020-12-10

**Authors:** Robert D. Huang, Lucienne Lutfy-Clayton, Douglas Franzen, Alexis Pelletier-Bui, David C. Gordon, Zachary Jarou, Jim Cranford, Laura R. Hopson

**Affiliations:** * University of Michigan, Department of Emergency Medicine, Ann Arbor, Michigan; † University of Massachusetts Medical School – Baystate Health, Department of Emergency Medicine, Springfield, Massachusetts; ‡ University of Washington School of Medicine, Department of Emergency Medicine, Seattle, Washington; § Cooper Medical School of Rowan University, Department of Emergency Medicine, Camden, New Jersey; ¶ Duke University, Department of Emergency Medicine, Durham, North Carolina; || St. Joseph Mercy Ann Arbor, Department of Emergency Medicine, Ypsilanti, Michigan

## Abstract

**Introduction:**

The average number of applications per allopathic applicant to emergency medicine (EM) residency programs in the United States (US) has increased significantly since 2014. This increase in applications has caused a significant burden on both programs and applicants. Our goal in this study was to investigate the drivers of this application increase so as to inform strategies to mitigate the surge.

**Methods:**

An expert panel designed an anonymous, web-based survey, which was distributed to US allopathic senior applicants in the 2017–2018 EM match cycle via the Council of Residency Directors in Emergency Medicine and the Emergency Medicine Residents Association listservs for completion between the rank list certification deadline and release of match results. The survey collected descriptive statistics and factors affecting application decisions.

**Results:**

A total of 532 of 1748 (30.4%) US allopathic seniors responded to the survey. Of these respondents, 47.3% felt they had applied to too many programs, 11.8% felt they had applied to too few, and 57.7% felt that their perception of their own competitiveness increased their number of applications. Application behavior of peers going into EM was identified as the largest external factor driving an increase in applications (61.1%), followed by US Medical Licensing Exam scores (46.9%) – the latter was most pronounced in applicants who self-perceived as “less competitive.” The most significant limiter of application numbers was the cost of using the Electronic Residency Application Service (34.3%).

**Conclusion:**

A substantial group of EM applicants identified that they were over-applying to residencies. The largest driver of this process was individual applicant response to the behavior of their peers who were also going into EM. Understanding these motivations may help inform solutions to overapplication.

## INTRODUCTION

In 2018 emergency medicine (EM) was the third most commonly matched specialty, comprising 7.5% of graduating allopathic seniors in the United States (US).^1^ A total of 1,748 US allopathic seniors ranked EM as their top specialty in the 2018 match with a mean contiguous ranking of 12.8 programs among matched applicants.^1^ In comparison to applicants from the 2016 match with regard to United States Medical Licensing Examination (USMLE) scores, experiences (research, volunteer, work), Alpha Omega Alpha Honor Medical Scoiety (AOA) status, and additional degree, the 2018 applicants were similar.^2^

Despite similarities in applicant characteristics, in just a two-year period the average number of US allopathic applications rose from 93,456 in 2016 to 111,964 in 2018, despite an increase of only 233 applicants.^3^ A greater number of applications requires a concurrent increase in time and effort by programs to review applicants and make decisions about interview selection.^4^ When coupled with the lack of robust outcomes data on which aspects of an applicant’s portfolio predict future residency success, program directors and coordinators must spend substantial resources attempting to analyze these applications in order to find those who may be a “best fit” for their program. Additionally, the increase in applications to EM residency puts additional financial strains on the applicants themselves.^5^ Students incur substantial financial costs from an increase in the number of residency applications and interviews on top of potentially substantial medical school debt. This occurs on top of the already expensive EM application process that values electronic Standardized Letters of Evaluation (eSLOE) from away rotations.^6^

Our objective in this study was to investigate the drivers of the increase in EM resident applications so as to inform potential strategies to mitigate the surge.

## METHODS

We created an anonymous, web-based survey for distribution to US allopathic senior applicants in the 2017–2018 EM match cycle. The author group represented a multi-institutional expert panel composed of academic EM faculty with both program and clerkship director experience to provide content validity. All survey designers had extensive experience with the match and application processes, represented diverse program formats and geographic regions, and had experience and expertise in survey design and survey-based research. We iteratively designed and refined the survey, which was piloted on a small group of first-year EM residents to obtain feedback on content and structure. This survey was disseminated using a survey-building tool (Qualtrics XM, Provo, UT) and was administered anonymously after the National Residency Match Program (NRMP) rank list certification deadline and prior to the release of match results in order to minimize response bias or a feeling of influence from the survey authors. The study was distributed via both the Council of Residency Directors in Emergency Medicine (CORD-EM) and the Emergency Medicine Residents’ Association (EMRA) listservs, as well as advertised on Twitter and the /r/medicalschool subreddit. To encourage participation, participants could elect to provide their email address on a separate unlinked survey for a gift card drawing. The study was given exempt status by the institutional review board of the lead author’s home institution.

**Population Health Research Capsule**

What do we already know about this issue?

While the number of individual applicants to emergency medicine (EM) increased by only 233 from 2016 to 2018, the number of overall applications increased by 18,508.

What was the research question?

What is driving the increase in EM resident applications?

What was the major finding of the study?

Individual applicant’s behavior was substantially motivated by the behavior of their peers going into EM.

How does this improve population health?

This project may inform future interventions by the EM community to create meaningful change in application behaviors.

In addition to demographic information, respondents were asked to give their perspective on multiple factors potentially influencing their application behavior, as outlined in Tables 2–4. The survey also asked for information on the number of programs applied to and factors influencing their decision. Additionally, respondents were asked to give their perspective on how multiple factors influenced the number of EM programs they applied to. Applicants were also asked to retrospectively evaluate whether they thought they had applied to too many, too few, or the right amount of EM programs.

The respondents were also broken into subgroups based on their self-assessment of competitiveness to evaluate for differentiation in trends among applicants who identified as “very competitive,” “competitive,” and “less competitive” for EM residency. In addition to descriptive statistics, associations between self-perceived competitiveness were tested with one-way factorial analyses of variance for continuous outcomes and chi-squared analyses for categorical outcomes. Statistically significant effects of perceived competitiveness were followed up with post-hoc between-groups comparisons using Tukey “honestly significant difference” tests for continuous outcomes, and pairwise tests between percentages for categorical outcomes. An alpha of .05 was used for all inferential analyses.

## RESULTS

We received 532/1748 (30.4%) survey responses from US-senior allopathic medical students applying to EM in the 2018 NRMP Match cycle. The demographics of respondents are shown in Table 1. These demographics were compared to “Charting Outcomes in the Match of US Allopathic Seniors,” a report released by the NRMP. The respondents’ mean Step 1 (231.6) and Step 2 (246.6) scores were similar to the national means of EM (233 and 247, respectively), as was the percentage of AOA students (14.5% in our cohort, 12.4% nationally). The average number of programs applied to per applicant was 49.1.

We performed an analysis to correlate the information provided by the students with their perceived competitiveness (Table 2). There were strong and statistically significant correlations between self-perceived competitiveness with estimated class rank, AOA status, USMLE Step 1 score, and USMLE Step 2 clinical knowledge (CK) score.

Information related to external factors that might have influenced applicant perspective on numbers of applications is represented in Table 3. We found that 61.1% of respondents reported that input from peers going into EM led to an increase in the number of applications submitted. USMLE scores were the next most likely external factor to increase application numbers (46.9%). Other factors surveyed showed minimal effects. The most variability in response was seen in the category “advice from EM faculty advisors”: 37.7% of respondents reported an increase in the number of applications; 26.1% reported a decrease; and 33.3% reported no effect from advice.

The results of personal factors relating to EM application numbers are summarized in Table 4. Electronic Residency Application Service (ERAS) cost drove a decrease in applications for 34.3% of respondents. Respondent self-assessment of personal competitiveness increased application numbers in 57.7%. Other personal factors did not have substantial effect on application numbers.

Applicant self-assessment of the number of applications they submitted showed that 47.3% of respondents reported, in retrospect, that they had applied to too many programs, while 40.9% felt they applied to the right number of programs. Only 11.8% believed they had applied to too few.

We performed subgroup analysis on students based on their self-perceived competitiveness. This information is available in Table 5. There was a strong and statistically significant association between “self-perceived competitiveness” and the “number of programs applied to in Emergency Medicine” (*F* (2, 504) = 84.4, *P*< .001); those who perceived themselves as “less competitive” applied to considerably more EM programs compared to those who self-perceived as “competitive” and “very competitive.” Students who self-assessed as “less competitive” were also statistically significantly more likely to indicate that they applied to too few EM programs (χ^2^[4] = 67.3, *P*<.001) and went on statistically significantly fewer EM interviews (*F*[2,504] = 27.7, *P*<.001).

In addition, compared to those who self-perceived as “competitive” and “very competitive,” those who perceived themselves as “less competitive” were statistically significantly more likely to increase the number of applications submitted for EM residency due to the influence of self-assessment of personal competitiveness (χ^2^[6] = 138.2, *P*<.001) and USMLE scores (χ^2^[6] = 90.1, *P*<.001); and statistically significantly less likely to decrease the number of applications submitted for EM residency due to the influence of personal geographic limitations (χ^2^[6] =22.8, *P* =.001). Results also showed that compared to those who self-perceived as “very competitive,” those who perceived themselves as “less competitive” were statistically significantly more likely to increase the number of applications submitted for EM residency due to the influence of the Visiting Student Application Service (VSAS)/Away rotation experience (χ^2^[1] = 12.1,*P*<.001); social media resources (χ^2^[1] = 8.2, *P* = .004); and having a faculty advisor in EM (χ^2^[1] = 5.3, *P* =.02). “Less competitive” students were statistically significantly more likely to indicate that ERAS cost was not relevant to the number of applications submitted for EM residency (χ^2^[1] = 6.8, *P* = .009).

Those who perceived themselves as “less competitive” were not statistically significantly more likely to increase the number of applications submitted for EM residency due to the influence of peers going into EM (χ^2^[6] = 8.1.0, *P* = .23), but those who perceived themselves as “very competitive” were statistically significantly less likely to endorse this item compared to those who perceived themselves as “competitive” (χ^2^[1] = 6.5, *P* = .01). Finally, there were no statistically significant associations between self-perceived competitiveness and the number of applications submitted for EM residency due to the influence of other factors listed.

## DISCUSSION

Nearly half of US-senior allopathic EM residency applicants felt they had applied to too many programs in the 2018 NRMP Match application cycle. Based on correlations with objective achievement measures in the ERAS application, EM applicants as a whole were able to stratify themselves into relative zones of competitiveness. While the subgroup of “very competitive” applicants was more likely to report a decrease in applications due to their self-perceived competitiveness, 57.7% of total respondents said their self-assessment of competitiveness led to an increase in their number of applications. As this percentage outstrips the number of respondents who self-perceived as “less competitive,” it suggests that even applicants who viewed themselves as “competitive” felt the pressure to increase application numbers.

The reason behind this phenomenon, and our finding that the most substantial driver of increase in applications is peers going into EM, has been explained by others in medical education through game theory, particularly the prisoner’s dilemma paradox.^7^–^10^ In the prisoner’s dilemma, because direct cooperation isn’t possible and the larger payoff is thus uncertain, two individuals demonstrate self-interest and choose an option that minimizes their personal risk. In the application process, this translates to applicants choosing to overapply to mitigate the risk to themselves should their colleagues overapply, which they presume will happen.

Another hypothesis to explain the overapplication phenomenon is prospect theory.^11^ Prospect theory is a behavioral model that explains how people decide between different options, or prospects, that involve risk and uncertainty. In broad strokes, prospect theory holds that people overweigh losses compared to gains and are therefore more willing to take risks (i.e., pay more money for the residency application process) to avoid losses (i.e., going unmatched), no matter how small the probability of loss.

Given that the motivation for deans of medical schools is that their medical students match successfully, we had thought this might lead to advice encouraging students to overapply. However, a quarter of respondents reported not consulting their Dean’s office at all, while another half reported no effect on their application numbers. This seems to suggest that applicants are instead relying primarily on EM departmental resources (e.g., clerkship directors, trusted faculty, etc.) for application recommendations.

“Less competitive” applicants were statistically significantly more likely to increase their number of applications based on EM faculty advisor advice. This could have represented appropriate advising: “less competitive” applicants should require more applications to obtain an appropriate number of interviews to increase their chances of matching. However, one-third of “competitive” and “very competitive” applicants also reported an increase in applications based on EM faculty advisor advice. This suggests the possibility that EM advisors are contributing to the cycle of overapplication via their individual advising practices.

We hypothesized that several other factors would potentially increase application numbers, but the effects of these were mixed in our study results. The eSLOE is a critical component of EM residency applications in which writers rank applicants in a variety of clinical and non-clinical domains and in regard to their overall competitiveness as a residency applicant.^12^ This element did not appear to affect the majority of respondents despite its importance, potentially because students are blinded to their individual eSLOEs. The standardized video interview (SVI) was not a significant factor in application numbers; further, after our survey the Association of American Medical Colleges decided not to continue the EM-based pilot of the SVI.^13^ Social media resources were also either not used or non-contributory in a majority of applicants, as were peers going into non-EM specialties.

The VSAS and away rotation experience did not have an effect on applicants as a whole. However, “less competitive” applicants showed a statistically significant increased likelihood to report that the VSAS and away rotation experience affected their application numbers. This could represent clerkship directors at away rotations providing appropriate feedback on the applicant’s performance, which could in turn have informed the number of EM programs to which applicants applied. Alternatively, the current expressions of frustration with the away-rotation process, which often relies on an application process for limited slots, may push less competitive students to feel more anxiety surrounding away rotations than those who perceive themselves as more competitive for EM. This anxiety may have influenced those in this subgroup to apply to more programs during the actual residency application process.

USMLE scores were a motivator for increased applications in almost half of respondents. USMLE scores, and Step 1 in particular, are heavily weighted in the residency selection criteria across specialties^14^ and have become the primary motivator of the undergraduate medical education learning environment.^15^ The students who viewed themselves as less competitive were more likely to view their USMLE Step scores as a reason to increase their number of applications. Students could have been using their USMLE scores as a surrogate for competitiveness, undervaluing other pieces of their application. Alternately, students could have viewed USMLE scores as the application item that program directors find to be the most important, despite ongoing efforts encouraging a more holistic application review.^16^ The recent announcement from the Invitational Conference on USMLE Scoring (InCUS) that USMLE Step 1 will go to a pass/fail scoring system^17^ could change this perspective; but Step 2 CK will remain a scored exam.

### Proposals for Improving Overapplication

Several proposals have been put forth by the EM community with the hope of decreasing residency application numbers. The CORD-EM Application Process Improvement Committee has developed the Emergency Medicine Applicant Tool of Common Hangups (EMATCH) to increase transparency to students on their competitiveness.^18^ However, if already competitive applicants feel the need to overapply because of normal human tendencies demonstrated by the prisoner’s dilemma and prospect theory, it is unlikely that we can depend on applicants to curb overapplication through recommendations and advisement alone. Change in behavior may require external forces.

Over a third of “competitive” and “very competitive” applicants reported that they applied to more programs after speaking with EM faculty advisors. This finding suggests that EM faculty may be a target area for improving EM overapplication. CORD’s Advising Students Committee has worked with EMRA to create an advising guide and several other resources for students to determine how competitive their applications to EM are. However, if faculty advisors provide conflicting advice, this could add to student anxiety over the application process and worsen overapplication. There may be more work to be done by the EM residency education community to provide resources to standardize faculty advising practices.

There may also be a need to place external limitations on overall application numbers. These external limitations may take a variety of forms from overt restriction to systematic barriers to limit applications through increased work or cost to the applicant. Proposals that have been made by the EM community include the following: preventing interview double-booking through the use of a centralized interview scheduling system; limiting all EM residency interviews to one particular day of the week to limit the number of possible interviews an applicant can attend; increasing application costs; and increasing the difficulty of residency program application by mandating that applicants write program-specific letters of intent, similar to the process in place for the otolaryngology match.^19^ These solutions may also potentially serve to benefit applicants with greater financial and temporal means. Even so, prospect theory states this intervention will not work; applicants will take the additional loss of money to avoid not matching, just as they will take on the added administrative burden of program-specific applications.

The ultimate, yet extreme, solution may revolve around limiting or sequencing the number of applications an applicant can submit. However, this solution may be disadvantageous to applicants with unique situations, such as participation in a couples match, particularly if the two specialties are not synchronized with application review and interview times. Another solution proposed by Berger and Cioletti includes changing the entire match process to several rounds (limiting the number of applicants during each round), rather than the current process of one round of match followed by the Supplemental Offer and Acceptance Program.^9^ This model is also currently being explored by the American College of Obstetrics and Gynecology via the American Medical Association Reimagining Residency Grants.^20^ Whipple et al ran a variety of computer simulation models of the otolaryngology match examining a preference-weighted application.^21^ They found that the use of student-provided preferences (the ability to select a limited number of programs as their “preferred programs”) decreased the gap in the number of interviews received by the most and least competitive applicants and allowed programs to review more applicants without resorting to metric-based screening. The potential implications of these proposals on both program and applicant require extensive exploration.

## LIMITATIONS

While on objective metrics our respondents appear to closely match the overall EM applicant pool and suggest a representative sample, we captured only 30.4% of US allopathic senior applicants in the 2018 cycle. The distribution strategy of our survey likely contributed to this. While the use of public forums and listservs and total anonymity may have allowed respondents to feel comfortable entering sensitive information, it precluded follow-up to increase survey capture of the polled population. Our small sample size could have introduced confounders based on the percentage of the applicant pool most likely to respond to the survey. In order to best invite honest reporting, no specific measures could be taken to prevent anonymous participants from taking the survey more than once, which presents an additional confounder.

In addition, the distribution through listservs may also have biased responses toward those groups most connected to these administrative resources and the recommendations offered through them. Additionally, our survey relied on subjective data (competitiveness in EM), which introduces a possible confounder based on the inaccuracy of self-assessment. When looking at the geographic data by census tracts of each respondent, there were signs of overrepresentation of certain areas of the country with East North Central Midwest, Middle Atlantic, and South Atlantic regions being over-represented and the East South Central, Mountain West, New England, West North Central Midwest, and Pacific West being under-represented.

While we obtained information on the number of interviews applicants performed, we did not obtain information about the number of interview offers they received. Any excess of interview offers received compared to interviews completed could have affected applicant self-assessment and provided insight into disparities between the competitiveness subgroups.

We broke our respondents into subgroups based on their self-perceived competitiveness to further the evaluation of trends in “less competitive,” “competitive,” and “very competitive” subgroups. Without information on the respondents’ eSLOEs or individual achievements that are very real contributors to the strength of an application,^22^ a true competitiveness assessment was impossible. The correlation of self-perceived competitiveness with USMLE Step 1 and Step 2 CK scores, AOA status, and estimated class rank provide some validity evidence to the accuracy of this perception, but with the recognition that these markers provide only one aspect of an applicant’s competitiveness.

## CONCLUSION

Our results suggest that individual applicant’s EM application behavior is substantially influenced by peers. While frustrating to programs and applicants, the logical framework behind each applicant’s decision to overapply is not unusual based on known game-theory models. The EM application process has created an environment that has fueled overapplication. External limitations to applications numbers may be needed to create meaningful change in EM residency applicant behavior.

## Figures and Tables

**Table 1:** Demographics (N = 532) of allopathic medical students in the United States applying to emergency medicine residency.

Variable	% or M (SD)
Gender	
Female	38.2
Male	61.7
Other	0.1
Race/ethnicity	
Asian, Native Hawaiian or Pacific Islander, American Indian or Alaska Native	12.5
Black or African American	4.1
Hispanic/Latino	5.2
White	68.4
Other or more than one race	7.3
No response or decline to answer	2.4
Estimated class rank^a^	
Lower third	13.9
Middle third	47.6
Upper third	38.5
Geographic area of medical school	
East North Central Midwest (IL, IN, MI, OH, WI)	22.7
East South Central (AL, MS, KY, TN)	1.9
Middle Atlantic (NJ, NY, PA)	22.4
Mountain West (AZ, CO, ID, MT, NM, NV, UT, WY)	4.3
New England (CT, MA, ME, NH, RI, VT)	5.3
Pacific West (AK, CA, HI, OR, WA)	7.7
South Atlantic (DC, DE, GA, FL, MD, NC, SC, VA, WV)	17.1
West North Central Midwest (IA, KS, MN, MO, ND, NE, SD)	7.3
West South Central (AR, LA, OK, TX)	11.3

USMLE Step 1 score	231.6 (17.6)
USMLE Step 2 CK score	246.6 (15.6)
Elected to the AOA Honor Society while in medical school? (% yes)	14.5
How would you rank your competitiveness as an applicant in emergency medicine?^b^	
Less competitive	12.4
Competitive	52.2
Very competitive	35.5
How many programs did you apply to in emergency medicine?	49.1 (23.2)
How many INTERVIEWS in emergency medicine did you go on?	13.2 (4.2)
How many EM programs did your main EM faculty advisor recommend that you apply to?	38.4 (14.3)

a For this variable, 4.3% of the data were missing.

b For this variable, 4.1% of the data were missing.

*SD*, standard deviation; *USMLE*, United States Medical Licensing Examination; *AOA*, Alpha Omega Alpha Honor Medical Society; *EM*, emergency medicine.

**Table 2:** Competitiveness analysis (N = 532).

Variable	Competitiveness self-identification			Total	P-value

	Less competitive	Competitive	Very competitive		
Estimated class rank					
Lower third	34 (54.0%)	32 (12.0%)	5 (2.8%)	71 (13.9%)	< .001
Middle third	28 (44.4%)	171 (64.3%)	44 (24.3%)	243 (47.6%)	
Upper third	1 (1.6%)	63 (23.7%)	132 (72.9%)	196 (38.4%)	
AOA status					
No AOA chapter	1 (1.6%)	10 (3.8%)	16 (8.8%)	27 (5.3%)	< .001
No	62 (98.4%)	247 (92.9%)	101 (55.8%)	410 (80.4%)	
Yes	0 (0.0%)	9 (3.4%)	64 (35.4%)	73 (14.3%)	
USMLE Step 1 score, mean (SD)	218.1 (14.2)	229.1 (15.8)	239.8 (17.2)	231.5 (17.6)	< .001
USMLE Step 2 CK score, mean (SD)	232.6 (12.6)	246.2 (13.0)	251.9 (16.9)	246.5 (15.6)	< .001

*USMLE*, United States Medical Licensing Examination; *AOA*, Alpha Omega Alpha Honor Medical Society;, clinical knowledge.

**Table 3:** How did each of the following factors influence you to change the number of applications submitted for emergency medicine residency? (N = 532).

Variable	Percentage responded			

	Increase	Decrease	No effect	Not used
Peers going into EM	61.1	5.2	31.1	2.6
USMLE scores	46.9	23.6	28.7	0.8
Faculty advisor in EM	37.7	26.1	33.3	2.8
VSAS/Getting and doing an away rotation	29.5	15.2	50.3	5.0
Dean/Student Affairs advisor	24.8	5.6	47.1	22.6
Social media resources	19.2	1.6	44.5	34.7
Formal online advising resources from EM	18.2	7.2	45.5	29.1
eSLOE(s) processes	16.2	6.4	62.1	15.4
Peers going into other specialties	15.4	2.4	71.7	10.6
Standardized video interview (SVI)	13.6	1.8	74.1	10.6

*USMLE*, United States Medical Licensing Examination; *EM*, emergency medicine; *VSAS*, Visiting Student Application Service; *eSLOE*, electronic standardized letter of evaluation.

**Table 4:** How did each of the following personal factors influence you to change the number of applications you submitted for emergency medicine residency? (N = 532).

Variable	Percentage responded			

	Increase	Decrease	No effect	Not used
Self-Assessment of personal competitiveness within specialty	57.7	18.0	22.8	1.6
Personal geographic limitations	17.6	26.7	46.3	9.4
Couples match or other significant other considerations	14.8	1.8	18.6	64.9
ERAS cost	0.6	34.3	56.3	8.8

*ERAS*, Electronic Residency Application Service.

**Table 5:** Subgroup analysis by allopathic medical students’ self-perceived competitiveness (N = 532).

Variable	Competitiveness self-identification^1^			Comparisons		

	Less competitive (n=63, 12.3%)	Competitive (n=266, 52.2%)	Very competitive (n=181, 35.5%)	P-value for LC vs. C	P-value for LC vs. VC	P-value for C vs. VC
How many programs did you apply to in EM?	75.7	50.9	37.4	<.001**	<.001**	<.001**
Percent indicating that they applied to “too few” EM programs.	36.7%	12.9%	1.7%	.003*	<.001**	.02*
How many INTERVIEWS in EM did you go on?	9.8	13.3	14.2	<.001**	<.001**	.02*
Percent indicating that **SELF-ASSESSMENT OF PERSONAL COMPETITIVENESS** influenced them to increase the number of applications submitted for EM residency.	93.3%	69.7%	27.7%	.04*	<.001**	<.001**
Percent indicating that **USMLE scores** influenced them to increase the number of applications submitted for EM residency.	81.7%	52.7%	26.6%	<.001**	<.001**	.001*
Percent indicating that **PERSONAL GEOGRAPHIC LIMITATIONS** influenced them to decrease the number of applications submitted for EM residency.	8.3%	25.4%	35.0%	.004*	<.001**	.03*
Percent indicating that **VSAS-EXPERIENCE OF GETTING & DOING AN AWAY ROTATION** influenced them to increase the number of applications submitted for EM residency.	41.7%	33.7%	19.2%	.24	<.001**	<.001**
Percent indicating that **SOCIAL MEDIA RESOURCES** influenced them to increase the number of applications submitted for EM residency.	28.3%	21.6%	12.4%	.26	.004*	.01*
Percent indicating that **FACULTY ADVISER IN EM** influenced them to increase the number of applications submitted for EM residency.	50.0%	37.9%	33.3%	.08	.02*	.32
Percent indicating that **ERAS-COST** was not relevant to the number of applications submitted for EM residency.	16.7%	6.4%	9.6%	.009*	.14	.22
Percent indicating that **PEERS GOING INTO EM** influenced them to increase the number of applications submitted for EM residency.	58.3%	66.3%	54.2%	.24	.58	.01*

1 n = 22 (4.1%) has missing data on this variable.

* p<.05.

** p<.001.

*EM*, emergency medicine; *LC*; less competitive; *C*, competitive; *VC*, very competitive; *USMLE*, United States Medical Licensing Examination; *VSAS*, Visiting Student Application Service; *ERAS*, Electronic Residency Application Service.
